# Genetic Diversity and Phylogeny of *Aedes aegypti*, the Main Arbovirus Vector in the Pacific

**DOI:** 10.1371/journal.pntd.0004374

**Published:** 2016-01-22

**Authors:** Elodie Calvez, Laurent Guillaumot, Laurent Millet, Jérôme Marie, Hervé Bossin, Vineshwaran Rama, Akata Faamoe, Sosiasi Kilama, Magali Teurlai, Françoise Mathieu-Daudé, Myrielle Dupont-Rouzeyrol

**Affiliations:** 1 URE-Dengue et autres Arboviroses, Institut Pasteur de Nouvelle-Calédonie, Réseau International Institut Pasteur, Noumea, New Caledonia; 2 URE-Entomologie Médicale, Institut Pasteur de Nouvelle-Calédonie, Réseau International Institut Pasteur, Noumea, New Caledonia; 3 UMR 9220, ENTROPIE, Institut de Recherche pour le Développement, Noumea, New Caledonia; 4 Pôle des Maladies Infectieuses et Emergentes, Institut Louis Malardé, Papeete, Tahiti; 5 Ministry of Health, Suva, Fiji; 6 Ministry of Health, Nuku'alofa, Kingdom of Tonga; 7 URE-Epidémiologie des Maladies Infectieuses, Institut Pasteur de Nouvelle-Calédonie, Réseau International Institut Pasteur, Noumea, New Caledonia; 8 UMR IRD 224-CNRS 5290-UM1-UM2, MIVEGEC, Institut de Recherche pour le Développement, Noumea, New Caledonia; United States Army Medical Research Institute of Infectious Diseases, UNITED STATES

## Abstract

**Background:**

The Pacific region is an area unique in the world, composed of thousands of islands with differing climates and environments. The spreading and establishment of the mosquito *Aedes aegypti* in these islands might be linked to human migration. *Ae*. *aegypti* is the major vector of arboviruses (dengue, chikungunya and Zika viruses) in the region. The intense circulation of these viruses in the Pacific during the last decade led to an increase of vector control measures by local health authorities. The aim of this study is to analyze the genetic relationships among *Ae*. *aegypti* populations in this region.

**Methodology/Principal Finding:**

We studied the genetic variability and population genetics of 270 *Ae*. *aegypti*, sampled from 9 locations in New Caledonia, Fiji, Tonga and French Polynesia by analyzing nine microsatellites and two mitochondrial DNA regions (CO1 and ND4). Microsatellite markers revealed heterogeneity in the genetic structure between the western, central and eastern Pacific island countries. The microsatellite markers indicate a statistically moderate differentiation (*F*_ST_ = 0.136; P < = 0.001) in relation to island isolation. A high degree of mixed ancestry can be observed in the most important towns (*e*.*g*. Noumea, Suva and Papeete) compared with the most isolated islands (*e*.*g*. Ouvea and Vaitahu). Phylogenetic analysis indicated that most of samples are related to Asian and American specimens.

**Conclusions/Significance:**

Our results suggest a link between human migrations in the Pacific region and the origin of *Ae*. *aegypti* populations. The genetic pattern observed might be linked to the island isolation and to the different environmental conditions or ecosystems.

## Author Summary

*Aedes aegypti* is the major arbovirus vector in the Pacific region. The spread of this mosquito in the different islands seems to be linked to human activities at the beginning of the twentieth century. Since 2010, occurrence of arbovirus outbreaks increased in this region, with the co-circulation of dengue, chikungunya and Zika viruses. The lack of vaccines and treatments for these pathogens led the health authorities to implement vector control measures. In this study, we present the genetic structure and the phylogenetic data obtained from the analysis of 270 *Ae*. *aegypti* collected in the Pacific region. The infestation of the islands seems to have American and Asian origins. The genetic structure of the vector populations indicates a differentiation of the mosquitoes between the western, central and eastern Pacific island countries and the specific island isolation context. This differentiation could be related to the different environmental conditions in each island country.

## Introduction

Dengue fever is the most prevalent arthropod-borne viral infection of humans in tropical and subtropical countries [1]. In the Pacific region dengue virus outbreaks have occurred regularly since World War II [2]. However, over the last 5 years, the arbovirus outbreak profile in the Pacific region has changed. Indeed, the predominant circulation of a single dengue virus serotype moved on to the co-circulation of several dengue serotypes, along with the emergence of chikungunya and Zika viruses [2–5].

Dengue, chikungunya and Zika are arboviruses transmitted to humans through the bites of mosquitoes belonging to the genus *Aedes*, subgenus *Stegomyia*. In the Pacific region, many of these vectors are endemic species members of the “*scutellaris*” group, which, according to Belkin [6], could have derived from a single original species unintentionally introduced by the first Austronesian navigators 1500 to 2000 years ago. Owing to the very particular conditions of this region including strict isolation and ecological differences between the islands, it underwent a speciation process that led to the separation into different species [6, 7]. The introduction of *Aedes aegypti* was more recent, this mosquito was first recorded in the Pacific in the late nineteenth and the early twentieth century [8]. At present, the main *Aedes* vectors are *Ae*. *aegypti*, *Aedes albopictus*, *Aedes polynesiensis* along with nine other *Aedes* potential vectors [8]. *Ae*. *aegypti* is present in most Pacific islands with the exception of Futuna and very few other isolated islands. Recently introduced, from South Asia into Western Pacific islands, *Ae*. *albopictus* is now established as far as the Kingdom of Tonga. *Ae*. *polynesiensis* is widespread in the Eastern part of Oceania, including Fiji, Samoa Islands, French Polynesia, and Pitcairn [9]. Regarding this distribution, *Ae*. *aegypti* is the most widespread arbovirus vector in the Pacific with its presence reported in a majority of islands. This vector is a domestic species, closely associated with human migrations and transportation, commerce and urbanization [10, 11].

Pacific islands have experienced intense population migrations since the early nineteenth century with the first wave of European colonization [12]. During the twentieth century, this migration continued due to the implementation of various business and industrial activities. Thus many Asian workers immigrated into New Caledonia, French Polynesia [13] and Fiji. Population flows between the different Pacific islands have always been observed. This immigration was highly influenced by the vehicular languages used, being more intense between French speaking islands or between English speaking islands [12]. The introduction of *Ae*. *aegypti* in different islands over time might be linked to the Pacific history of human migrations. In French Polynesia it was first reported in 1924 only in Tahiti, and then in the Marquesas Islands and in the Austral Islands sixty years later [14] but the first dengue epidemic was described in the middle of the nineteenth century [15] and might be due to *Ae*. *polynesiensis* which is a competent dengue vector [16, 17]. The first reported dengue epidemic in New Caledonia was described during the 1880s. This epidemic episode clearly demonstrates the presence of *Ae*. *aegypti*, as no other dengue vector had been reported at this date or later on [18]. In Fiji and Tonga, vector descriptions reported the presence of *Ae*. *aegypti* from the 1960s [6, 19], but dengue epidemics were recorded before the 1950s [2]. During World War II, the exchanges between America, Asia, Europe and the Pacific islands increased and may have impacted the distribution of *Ae*. *aegypti* [20].

Before 1960, no systematic control measures were implemented against *Ae*. *aegypti* in the South Pacific islands, except for international airports and harbors [19]. Due to an increase in the frequency and intensity of dengue outbreaks in the second half of the twentieth century, French Polynesia and New Caledonia health authorities adopted similar vector control strategies involving a combination of insecticide spraying and community awareness raising, aimed at source reduction. These strategies resulted in a decrease of the mosquito’s presence in these island groups [21–23]. In 2003, in Fiji, the Ministry of Health decided to assess a larval source reduction campaign to reduce the density of the vector’s breeding sites [24]. In Tonga the WHO decided in 1984 to increase the vector control effort at the international airport with insecticide applications and aircraft disinsection. These vector control operations did not result in elimination of *Ae*. *aegypti*, but they created different environments and exerted selective pressure.

To our knowledge, no studies have investigated the genetic diversity of *Ae*. *aegypti* in the Pacific region except in French Polynesia, using isoenzymes [11] and alloenzymes [25]. These studies demonstrated a link between the genetic diversity of *Ae*. *aegypti* populations, human population density, and vector control intensity. The recent arbovirus outbreak waves in the Pacific region highlight the need to improve our knowledge of *Ae*. *aegypti* in the Pacific. The aim of this study is to better understand the genetic structure and the phylogeny of this vector on the Pacific region. For this purpose, we analyzed a set of nine microsatellites and two mitochondrial DNA sequences on 270 *Ae*. *aegypti* specimens collected in nine locations distributed in four different Pacific Island Countries and Territories.

## Materials and Methods

### Description of the study area

**Fig 1:**
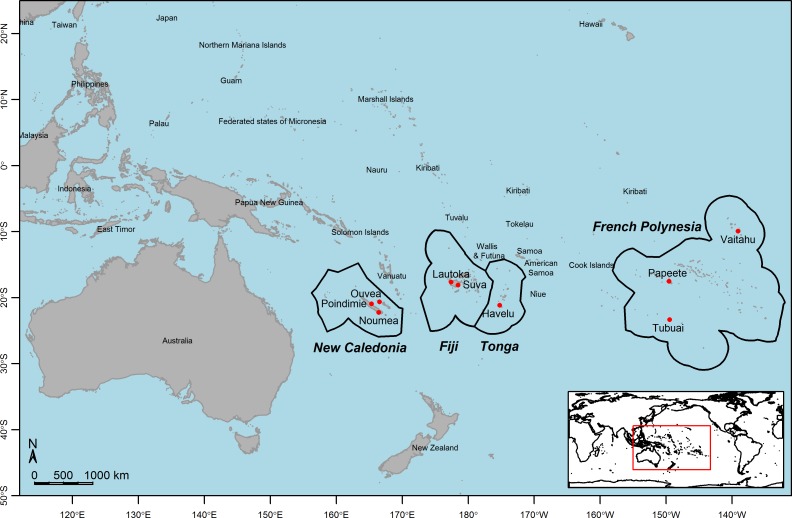
Pacific map locating *Ae*. *aegypti* sampling sites, 2013. The nine sample sites are represented by the red dots.

The Pacific region is an area unique in the world, composed of thousands of islands, high volcanic and low coral (atoll) islands, separated by vast stretches of ocean. Our sample sites are situated between longitudes 165° East and 139° West, spanning a region approximately 6,000 km wide. Latitudes of our sample sites are between 9° South and 23° South. There are several tropical climatic zones across the South Pacific region with different environments according to the latitude, localization within the islands and human influence. In New Caledonia (NC), temperatures are generally mild although with marked seasons. Poindimie, situated in a rural area exposed to dominant winds, has heavy rainfall whereas Noumea, the main city, is much drier. Ouvea is a flat coral island with no water supply connection (Fig 1). In Fiji (FJ), both sample sites are situated in peri-urban settings. Lautoka, a city on the leeward side of the island, is situated in a dry area. Suva, on the windward side of the same island, has a wet climate. In Tonga (TG), Havelu is a suburb of Nuku’alofa, capital of the country, on the island of Tongatapu, a flat coral island, but, where piped water supply is available, unlike on Ouvea (Fig 1). In French Polynesia (FP) the climate is different between the islands. Tubuai is the southernmost sample site of all. It is a rural island with temperate climate and distinct seasons. Papeete is the main city on Tahiti, with a humid tropical maritime climate and high temperature with slight seasonal variations. The village of Vaitahu on the island of Tahuata in the Marquesas Islands, is the northernmost sample site with warm conditions year round (Fig 1).

### Mosquito sampling

Mosquitoes were sampled at the immature stage (larvae and pupae) in the four island countries: New Caledonia (NC, 3 sites), French Polynesia (FP, 3 sites), Fiji (FJ, 2 sites) and Tonga (TG, 1 site) (Fig 1). For each sampling site a central spot was specified (Table 1). All potential breeding sites within a 200 m radius were searched and mosquito larvae and pupae were collected (three to eleven containers were sampled per site). A first morphological identification was carried out. *Aedes*-like larvae were reared to adulthood for confirmation and the *Ae*. *aegypti* specimens collected. Thirty such specimens from each site were stored in 100% ethanol at -20°C for molecular analysis.

**Table 1:** *Ae aegypti* sampling sites: coordinates and date in Pacific islands, 2013.

Sample name	Locality	Country	Date	Latitude	Longitude
**Poi-NC**	Poindimie	New Caledonia	Jul. 2013	20°56'56" S	165°19'58" E
**Nou-NC**	Noumea	New Caledonia	Jul. 2013	22°13'57" S	166°25'25" E
**Ouv-NC**	Ouvea (Loyalty Islands)	New Caledonia	Jul. 2013	20°39'00" S	166°32'38" E
**Lau-FJ**	Lautoka (Viti Levu Island)	Fiji	Oct. 2013	17°39'33" S	177°24'17" E
**Suv-FJ**	Suva (Viti Levu Island)	Fiji	Oct. 2013	18°05'13" S	178°27'43" E
**Hav-TG**	Havelu (Nuku’alofa Tongatapu Island)	Tonga	Oct. 2013	21°09'03" S	175°13'12"W
**Tub-FP**	Mataura (Tubuai Australes Islands)	French Polynesia	Nov. 2013	23°20'49" S	149°28'43" W
**Pap-FP**	Papeete (Tahiti)	French Polynesia	Aug. 2013	17°31'38" S	149°33'00" W
**Vai-FP**	Vaitahu (Tahuata Marquisas Islands)	French Polynesia	Jan. 2013	9°56'14" S	139°06'29" W

Thirty mosquitoes were analyzed for each sampling site.

### DNA extraction

Total DNA was extracted from adult mosquitoes using the DNeasy Blood & Tissue Kit (Qiagen) with a first step of mechanic lysis with 2.38 mm RNase/DNase free metal beads at 3000 rpm during 1 min and stored at -20°C.

### Microsatellite analysis

Individual genotypes were scored for 11 previously published microsatellite loci: AC1, AC2, AC4, AG1, AG2, AG5, CT2 [26], A1, B2, B3 [27] and 145TAAA1 [28]. DNA was amplified in a Veriti 96 well Thermal Cycler (Applied Biosystems) using the GoTaq G2 Flexi DNA Polymerase (Promega) as described in previous studies with slight modifications [26–28]. PCR products were analyzed using a Genetic Analyser 3130xl (Applied Biosystems). The results were genotyped with Peak Scanner software (Applied Biosystems) and double-checked (i. e., read by two independent people).

The deviation from Hardy-Weinberg equilibrium for each locus was tested with GenAlex 6.5 [29]. MicroChecker v2.2.3 [30] was used to calculate the probability of null allele occurrence in each locus within each population. The number of alleles and the estimated allele richness [31] were determined by FSTAT2.9.3 software [32]. The *F*_IS_ for each population for all loci were calculated using Genetix [33] and Arlequin v3.5.1.2 [34]. The Analysis of Molecular Variance (AMOVA) and the *F*_ST_ were computed using Arlequin v3.5.1.2 software [34]. The population genetic structure was determined using STRUCTURE software [35]. The Bayesian approach was chosen to infer the number of genetic clusters (K). We performed twenty independent runs, K from 1 to 15, with a burn-in period of 100,000 iterations and a total of 1,000,000 Markov Chain Monte Carlo iterations. The program Structure Harvester [36] was used to determine the most probable number of clusters by calculating the ΔK value [37]. The web server CLUMPAK was used to summarize and visualize the STRUCTURE results [38]. A Mantel test of correlation between geographical and genetic distance matrices was tested on IBD web server 3.23 [39] with 1,000 permutations.

### MtDNA sequencing analysis

A mitochondrial DNA analysis was performed for two genes: CO1 [40] and ND4 [41]. The 270 DNA samples were amplified and sequenced with the primers previously published. Amplified fragments were purified with the MinElute PCR Purification kit (Qiagen) and sequenced using BigDye Terminator v3.1 Cycle Sequencing kit (Applied Biosystems) on a Genetic Analyser 3130xl (Applied Biosystems).

Sequences were analyzed using Staden Package (MRC Cambridge, England), nucleotide sequences were aligned with BioEdit [42]. The haplotype numbers were assigned in reference to the published *Ae*. *aegypti* sequence from Cambodia for CO1 (GenBank accession No. JQ926688) and for ND4 (GenBank accession No. JQ926722). The nucleotide diversity (π), the Tajima [43], the Fu and Li [44] and Fu [45] tests were computed by DNASP v5 [46] to determine the neutrality of the populations. The phylogenetic networks based on CO1 and ND4 sequences were constructed using a reduced-median algorithm [47] as implemented in the Network program [48]. MRBAYES 3.1.2 software [49] was used to make a CO1-ND4 combined analysis using sequences obtained in this study and retrieved from GenBank. Four Markov chains were run for 2,000,000 generations with a 25% burn-in. The tree was drawn with FigTree v1.4.2 (Institute of Evolutionary Biology, University of Edinburgh). A Principle Coordinate Analysis (PCoA) of the mtDNA sequences was realized with DARwin software [50].

## Results

### Microsatellites analysis

#### Genetic variability of the samples

The 11 microsatellite loci allowed the identification of genotypes from the 270 *Ae*. *aegypti* adult mosquitoes sampled from the nine sites. The presence of a null allele was suspected at loci AG2 and 145TAAA1, which were therefore excluded from the analysis, leading to a dataset of 9 loci. A total of 57 alleles was observed for all samples (Table 2). The allelic richness was determined as 6 for the whole population ranging from 4 alleles for locus AC2 to 9 alleles for locus AG5. Regarding the allelic richness, no significant difference was observed among the different sampling sites or the different island countries.

**Table 2:** Genetic variability parameters estimated for the 9 microsatellites markers analyzed for all mosquito samples.

	AC1			AC2			AC4			A1			AG1			AG5			CT2			B2			B3			All Loci		
	*Nall*	*Rs*	*Fis*	*Nall*	*Rs*	*Fis*	*Nall*	*Rs*	*Fis*	*Nall*	*Rs*	*Fis*	*Nall*	*Rs*	*Fis*	*Nall*	*Rs*	*Fis*	*Nall*	*Rs*	*Fis*	*Nall*	*Rs*	*Fis*	*Nall*	*Rs*	*Fis*	*Nall*	*Rs*	*Fis*
Poi-NC	6	6	0,015	4	4	-0,055	4	4	-0,065	3	3	0,376	5	4,967	0,348	4	4	-0,08	2	2	0,223	5	4,967	0,173	3	3	-0,023	**36**	**4**	**0,086**
Nou-NC	6	6	-0,011	4	3,966	-0,074	4	4	-0,076	3	3	0,095	5	5	0,038	5	5	-0,12	2	2	0,262	2	2	0,721	2	2	-0,324	**33**	**3,7**	**0,045**
Ouv-NC	4	3,967	0,139	3	3	-0,236	5	4,966	-0,052	3	3	0,206	5	6,933	0,051	5	4,999	0,059	2	2	0,296	3	2,967	-0,065	3	3	-0,108	**33**	**3,7**	**0,017**
Lau-F	6	5,967	0,01	3	3	0,033	2	2	-0,177	5	4,966	-0,099	4	4	0,025	7	6,999	0,132	4	3,967	-0,113	4	4	-0,09	3	3	-0,295	**38**	**4,2**	**-0,055**
Suv-F	5	4,999	-0,229	4	4	-0,117	2	2	-0,387	5	4,967	-0,031	4	4	0,146	6	5,966	-0,08	3	2,967	0,138	4	4	-0,386	4	4	-0,204	**37**	**4,1**	**-0,128**
Hav-T	6	5,933	0,158	4	3,967	0,119	2	2	0,141	5	4,966	-0,135	3	3	0,162	4	4	0,136	4	4	-0,152	4	3,966	-0,67	5	4,967	0,186	**37**	**4,1**	**0,009**
Mat-FP	4	4	-0,162	4	3,967	0,056	2	2	0,102	4	3,999	0,037	4	4	0,03	5	5	-0,087	2	2	-0,057	3	3	0,127	3	3	-0,092	**31**	**3,4**	**-0,013**
Pap-FP	4	4	0,165	4	3,967	0,157	2	2	-0,208	4	3,999	-0,038	4	4	0,107	5	5	0,113	3	3	-0,101	5	4,967	-0,218	4	3,967	-0,205	**35**	**3,9**	**-0,004**
Vai-FP	4	4	0,075	3	3	0,238	2	2	-0,137	2	2	0,125	5	5,965	-0,188	5	5	0,045	4	3,933	-0,083	5	5	-0,072	2	2	0,065	**32**	**3,6**	**0,029**
New Caledonia	6	5,983	0,145	4	3,973	-0,106	5	4,333	-0,048	3	3	0,261	5	5,665	0,169	6	5,785	0,039	2	2	0,283	5	4,235	0,286	3	2,999	-0,103	**39**	**4,3**	**0,103**
Fiji	6	5,5	-0,099	4	3,994	-0,024	2	2	-0,216	5	4,751	-0,061	4	3,994	0,1	8	6,995	0,055	5	3,997	0,033	5	4,878	-0,137	4	3,941	-0,249	**43**	**4,8**	**-0,062**
Tonga	6	5,933	0,158	4	3,967	0,119	2	2	0,141	5	4,966	-0,135	3	3	0,162	4	4	0,136	4	4	-0,152	4	3,966	-0,67	5	4,967	0,186	**37**	**4,1**	**0,009**
French Polynesia	4	4	0,082	4	3,999	0,237	2	2	-0,094	5	4,113	0,17	5	5,114	0,059	7	6,023	0,143	4	3,333	0,074	5	4,791	-0,005	4	3,788	0,092	**40**	**4,4**	**0,099**
All samples	8	7,985	0,123	4	4	0,142	5	4,993	0,104	6	6	0,228	6	8,985	0,172	9	9	0,129	8	7,989	0,131	6	6	0,061	5	5	-0,021	**57**	**6,3**	**0,12**

N*all* corresponds to the number of scored alleles; R*s* represents the Allele Richness; *F*_IS_ indicates the Inbreeding coefficient.

### Genetic structure of the samples

**Fig 2:**
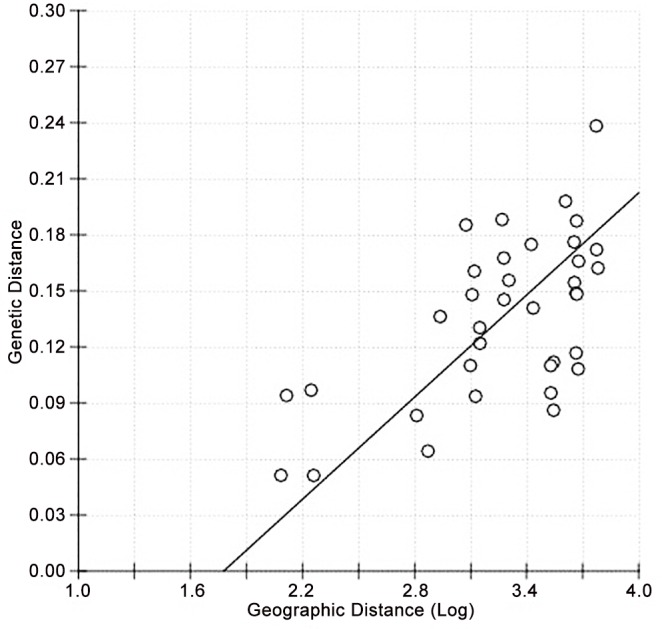
Correlation between the geographic and genetic distance matrices. The regression line corresponds to the standard major axis regression between pairwise genetic distances and logarithmic geographic distances with equation: Fst = - 0.1620 + 0.09113·log (geographic distance). The relationship was significant (Mantel test: Z = 16.3746; r = 0.6164; P < 0.001).

The AMOVA results indicated statistically moderate genetic differentiation for all samples (*F*_ST_ = 0.136; P < = 0.001). *F*_ST_ among the studied sample sites ranged from 0.05 to 0.24 (Table 3). The highest *F*_ST_ value was obtained between Ouvea (NC) and Vaitahu (FP) with 0.24. The two lowest results were obtained between Suva (FJ) and Lautoka (FJ) on one hand, while between Poindimie (NC) and Noumea (NC) on the other hand with a score of 0.05. Globally the *F*_ST_ results for Vaitahu (FP) were higher than the others (ranging from 0.13 to 0.24). Moreover, statistically high differences were observed between the New Caledonia samples and the samples of central (FJ and TG) and eastern Pacific (FP). The results of the Mantel test demonstrated a significant correlation between the genetic differentiation and the geographical distance (r = 0.6164; P < 0.001) (Fig 2) for the Pacific samples analyzed.

**Table 3:** Pairwise *F*_ST_ values for the nine populations studied and geographic distances between the sampling sites (in km).

	Poi-NC	Nou-NC	Ouv-NC	Lau-FJ	Suv-FJ	Hav-TG	Tub-FP	Pap-FP	Vai-FP
**Poi-NC**	-	182	130	1318	1412	2017	4643	4737	6050
**Nou-NC**	0.051	-	176	1255	1338	1899	4505	4618	5946
**Ouv-NC**	0.094	0.097	-	1188	1282	1894	4527	4613	5923
**Lau-FJ**	0.161	0.110	0.185	-	122	865	3499	3498	4763
**Suv-FJ**	0.122	0.094	0.148	0.051	-	744	3378	3383	4656
**Hav-TG**	0.156	0.145	0.168	0.136	0.064	-	2657	2720	4053
**Tub-FP**	0.187	0.176	0.154	0.112	0.110	0.175	-	647	1854
**Pap-FP**	0.108	0.117	0.149	0.087	0.095	0.141	0.083	-	1408
**Vai-FP**	0.162	0.172	0.238	0.166	0.148	0.198	0.188	0.130	-

Below diagonal, *F*_ST_ values, statistical significance was 0.05. Above diagonal, geographical distances (km) between the sample sites.

**Fig 3:**
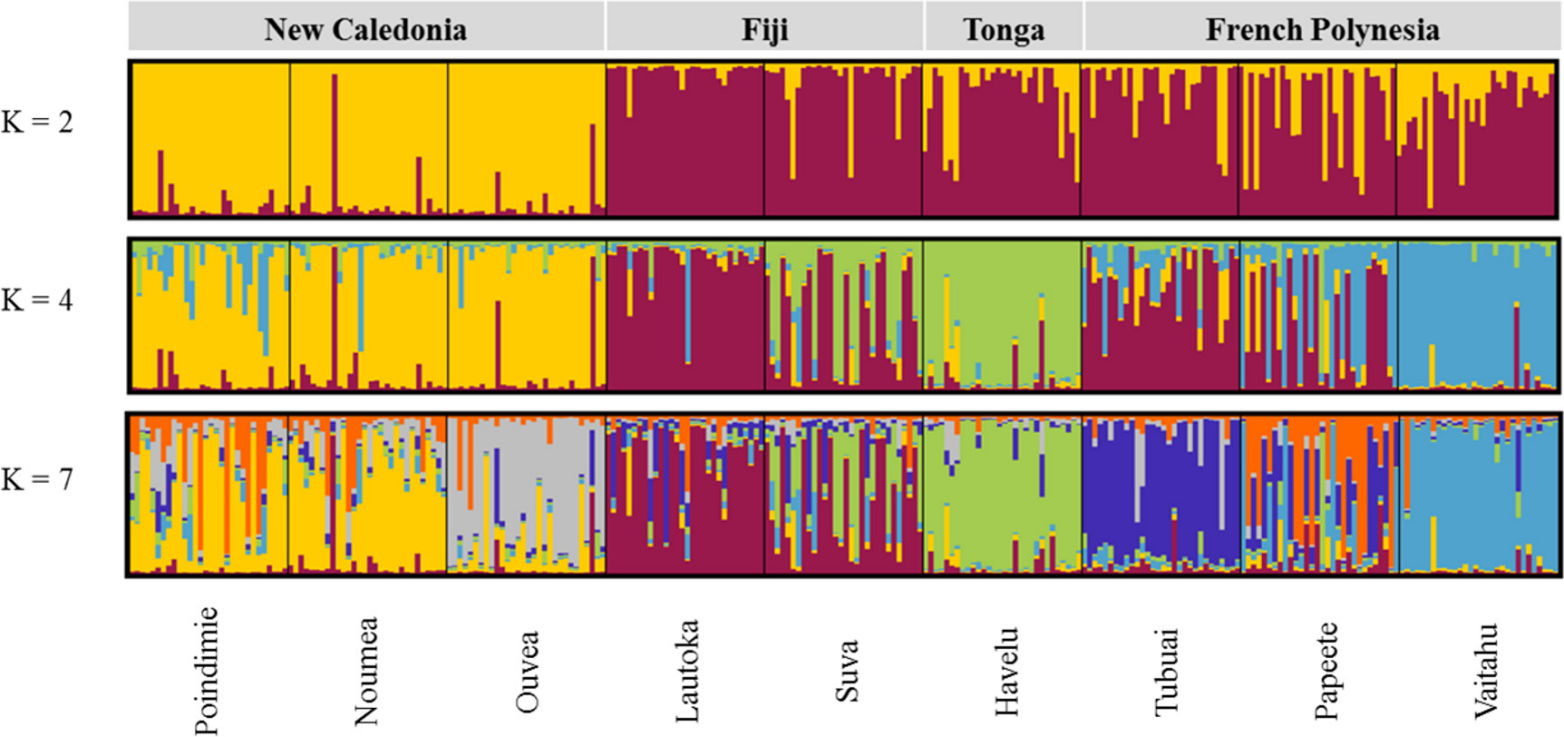
Model-based clustering of 270 *Ae*. *aegypti* individuals using STRUCTURE software. Each individual is represented by a single vertical line; sample sites are separated by a black line; the whole sample is divided into K colors representing the number of clusters assumed. The colors show the estimated individual proportions of cluster membership.

The Bayesian analysis performed, with the Evanno *et al* method [37], revealed that the most likely number of clusters were K = 2, K = 4 (highest probability) and K = 7. These three clustering models were further analyzed. The two-cluster plot (K = 2) indicates a differentiation between the samples of New Caledonia (West Pacific) on one hand, Fiji, Tonga (Central Pacific) and French Polynesia (East Pacific) on the other hand (Fig 3). The four-cluster plot (K = 4) highlights a differentiation between the samples of Fiji and Tonga. The Suva samples seemed to be more differentiated than the Lautoka or Havelu samples. For French Polynesia the Vaitahu samples appeared to be a separate cluster and the Papeete individuals looked to be more diversified than the other FP samples. The seven-cluster plot (K = 7) confirmed the results obtained with the AMOVA: a high diversity for Suva and Papeete, mid-diversity for Noumea/Poindimie and Lautoka and isolation of Ouvea, Havelu, Tubuai and Vaitahu.

### MtDNA phylogeny analysis

#### CO1 gene diversity

**Fig 4:**
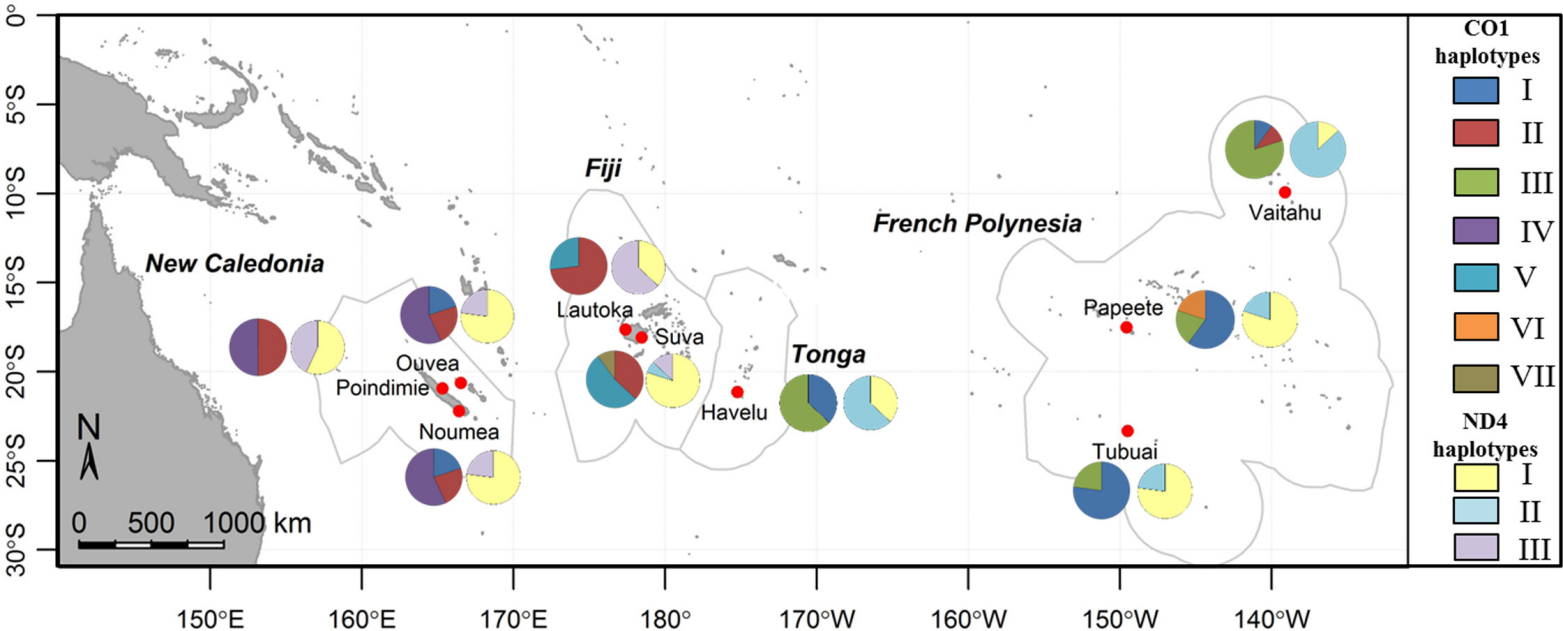
Representation of the MtDNA haplotype frequencies within the sample sites. The left circles indicate the CO1 haplotype frequencies and the right circles the ND4 haplotype frequencies. The arc length of each slice is proportional to the haplotype frequencies (as an example a semicircle represents 15 samples). Haplotype frequencies are indicated in S1 Table.

**Fig 5:**
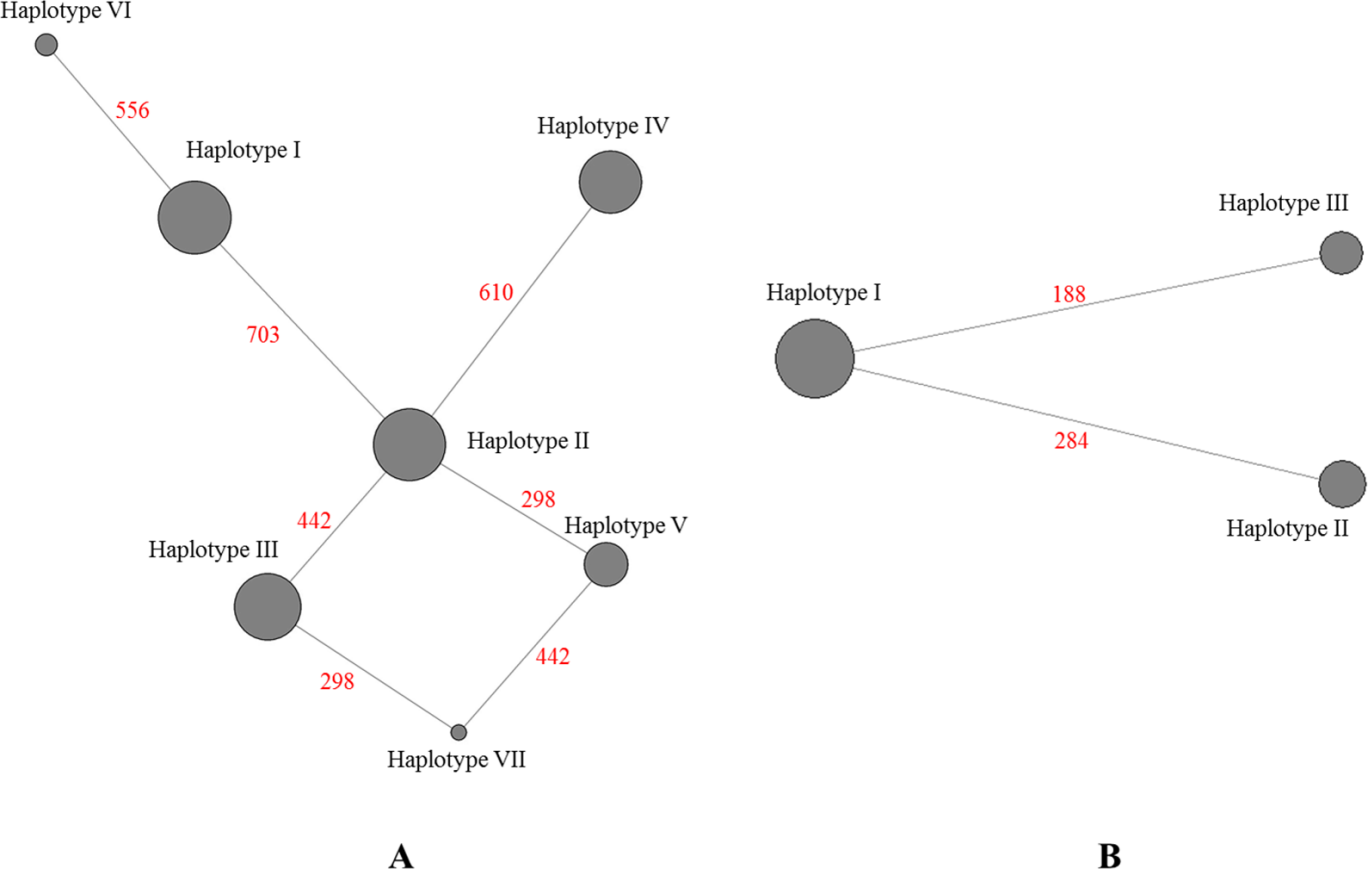
Median-joining network obtained with the haplotypes of all samples. A- Representation for the mtDNA CO1 sequences. B- Representation for mtDNA ND4 sequences. The diameters of grey circles represent the frequency of each haplotype for all individuals. The red number indicates the position of the mutation on the analyzed sequences.

All 270 individuals were analyzed for their CO1 sequence. The 711 bp alignment revealed the presence of seven distinct haplotypes (Figs 4, 5 and S1 Table, S2 Table) and a nucleotide diversity of π = 0.00177. Haplotype I (frequency = 0.25) was present in all islands except in Poindimie (NC) and in Fiji. Haplotype II (frequency = 0.24) was present in New Caledonia, Fiji and Vaitahu (in FP). Haplotype III (frequency = 0.21) was found in eastern islands (FP). Haplotype IV (frequency 0.18) seemed to be exclusively present in NC. The haplotype V (frequency = 0.09) was present only in Fiji. Haplotype VI (frequency = 0.02) was present only in Papeete (FP). And haplotype VII (frequency = 0.01) appeared to be exclusive to Suva (FJ). Haplotype II seemed to be the link between the different CO1 haplotypes in the Pacific region (Fig 5A). Haplotype VI appeared to derive from haplotype I with a single mutation. A link between haplotype VII and haplotypes III and V seemed to be present. Tajima’s D statistic (D = 1.02758, P > 0.10), used to determine the departure from neutrality, was not significant but suggested a balancing selection or a decrease in population size due to the presence of multiple alleles, some at low and other at high frequencies. Fu and Li’s statistics were positive but not significant (F* = 1.17029, P > 10; D* = 0.95715, P > 0.10) and confirmed the Tajima’s D result.

#### ND4 gene diversity

Partial ND4 sequence was analyzed for all individuals (Figs 4, 5 and S1 Table, S2 Table). The 320 bp alignment revealed the presence of three distinct haplotypes and a nucleotide diversity of π = 0.00203. Haplotype I (frequency = 0.59) was present in all Pacific samples. As for haplotype II (frequency = 0.22) the results demonstrated its presence only in central and eastern islands from Suva to Vaitahu. Haplotype III (frequency = 0.19) was present in the western islands from Poindimie (NC) to Suva (FJ). Haplotypes II and III seem to derive from haplotype I with only a single nucleotide difference (Fig 5B). The departure from neutrality, indicated by Tajima’s D statistic, was not significant (D = 1.131386, P > 0.10). Fu and Li’s statistics were positive but not significant either (F* = 0.99728, P > 0.10; D* = 0.61853, P > 0.10).

#### Associated CO1-ND4 sequences analysis

**Fig 6:**
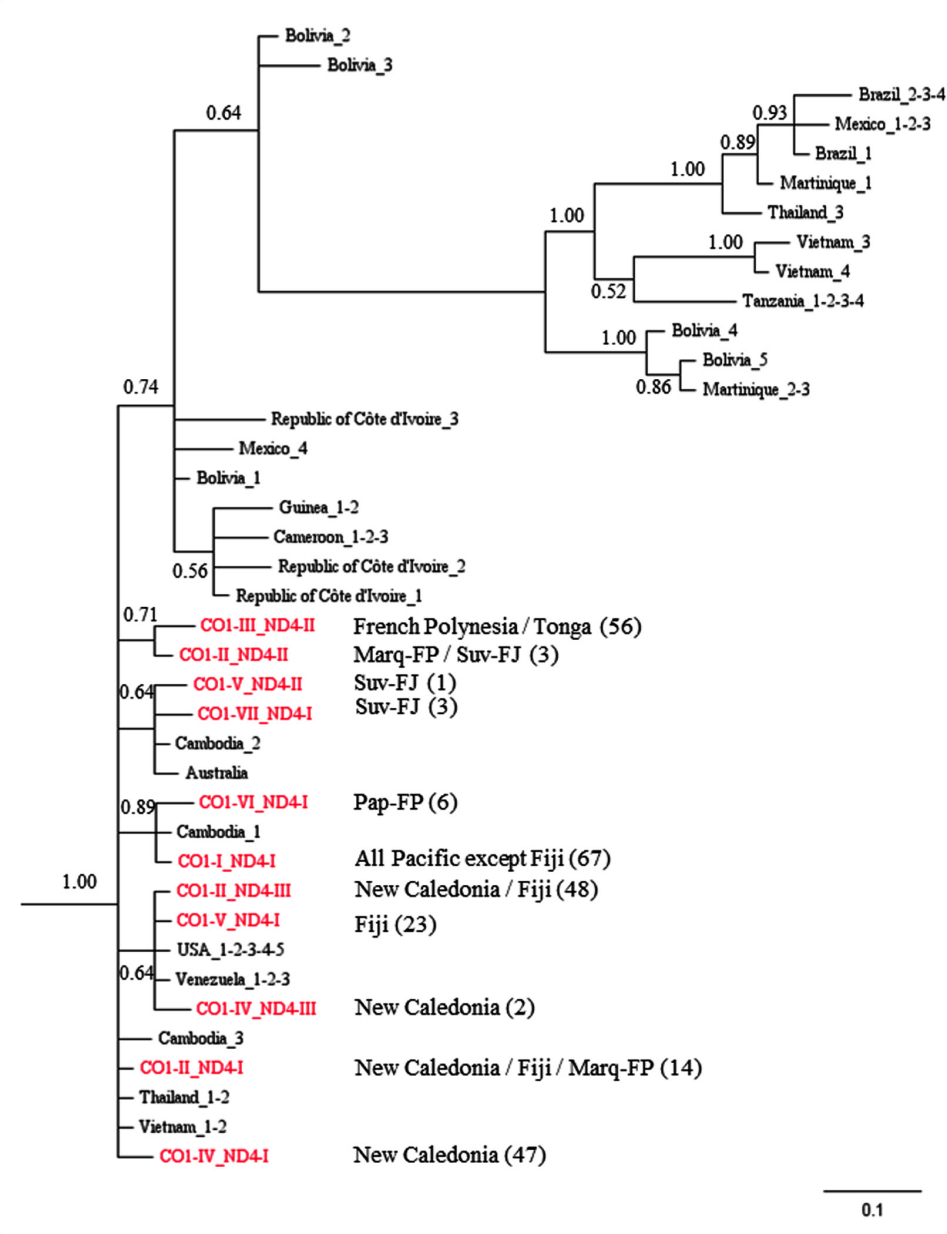
Phylogenetic tree obtained with a Bayesian inference of concatenated CO1 and ND4 sequence data. Numbers in parentheses indicate the number of samples belonging to this haplotype. For the Australian sample, only the CO1 sequence was available. Rooting was inferred from DNA sequences of *Anopheles pullus* and *Culex quinquefasciatus* but were not represented for clarity

The sequences of CO1 and ND4 were concatenated to perform a phylogenetic analysis with published mtDNA sequences from extra-Pacific *Ae*. *aegypti* specimens (Fig 6 and S3 Table). The phylogenetic tree obtained indicated the presence of a main combined haplotype in Pacific samples (CO1-I / ND4-I) originating from Asia. Specimens from the West Pacific (NC and FJ) were both linked to American mosquitoes and to Asian mosquitoes. A haplotype of Papeete-FP originated from Asia. Two haplotypes of Suva-FJ seemed to be linked to Australian specimens (CO1 mtDNA) and Asian specimens. The other samples principally of French Polynesia, Tonga and Suva-FJ were not clearly affiliated. The PCoA performed on associated CO1-ND4 sequences (S1 Fig) corroborated the results obtained. In fact, it underlined three origins: Asian, American and Australian.

## Discussion

The spread of *Ae*. *aegypti* in the Pacific took decades [6, 14, 18, 19]. The presence of this vector contributed to an explosive arbovirus situation in the Pacific region since the year 2010. Pacific Island Countries and Territories tried to limit the importation of *Ae*. *aegypti* in new territories through the establishment of control measures at sea-ports and airports. These measures may have prevented the increase of mosquito populations, but no evidence of eradication of the vector was ever recorded as it was in South American or Mediterranean countries [40, 51, 52]

Our results indicate the presence of multiple clusters in the mosquito samples from the Pacific islands [40]. Our *Ae*. *aegypti* samples are linked to *Ae*. *aegypti* originating from the Americas, South-East Asia and Australia (Fig 6 and S1 Fig). The introduction of the Asian lineage in New Caledonia could be linked to i) whaling industry and the sandalwood commerce between China/Australia/Pacific (Fiji and New Caledonia) in the years 1800–1850 [53] and ii) the immigration of Asian workers to New Caledonia during the years 1900–1940 for the mining industry [54]. In French Polynesia the presence of the Asian lineage could be linked to a substantial immigration of Chinese people, with three waves of immigration: the first one in 1865, then from 1907 to 1914 and lastly from 1921 to 1925 [13]. The presence of *Ae*. *aegypti* of the American origin in the Pacific could be associated with the whaling activity during the XIXth century, and to the presence of the US navy during World War II. From 1942 to 1945, New Caledonia was a support base and a key location for the US and Allied troops fighting in the Pacific War. The NC human population doubled during this period, and a dramatic increase in trade and economic activity took place. Furthermore, between the years 1880 and 1910, most importations into New Caledonia came from Australia, the United States and France with at least one ship calling at Noumea every two days [55]. In Fiji, the link with the Australian cluster could be related to the sugar cane and sandalwood trade between the two countries [53]. The principal commercial exchanges of Fiji, where a large proportion of the current population is of Indian origin, were with India, South-East Asia, Australia, the United States, and Europe [55]. The same commerce pattern is valid for Tonga. It is important to note that although this *Ae*. *aegypti* migration through the Pacific started during the nineteenth century, it might still be a current phenomenon. *Ae*. *aegypti* was first recorded in New Caledonia’s outer islands Mare and Lifou only in the 90’s and on Isle of Pins in 2003 [56]. In French Polynesia, *Ae*. *aegypti* was first recorded in the Austral Islands in 1984 [14]. On another hand, *Ae*. *albopictus* is currently invading the Pacific region, it has been reported in Fiji in 1988 [57], in Tonga in 2011 [58] and in Vanuatu in 2012 [59]but not yet in French Polynesia and New Caledonia [8, 60].

The presence of mitochondrial pseudogenes was observed in Fiji mosquitoes (two samples for Suva and one for Lautoka) for CO1 mtDNA (Haplotype CO1-V and CO1-VII, Fig 5 and S2 Table) with difficult distinction between a C and a T nucleotide. The presence of pseudogenes has already been demonstrated in *Ae*. *aegypti* nuclear genome. This genetic phenomenon, called heteroplasmy, was highly prevalent in previous studies [61, 62]. Among all the populations studied, it is interesting to note that this heteroplasmy was found only in Fiji samples, where haplotype distribution from eastern and western Pacific overlap, thus suggesting that Fiji might act as a hub regarding *Ae*. *aegypti* diversity.

In general, the genetic diversity observed within the Pacific was lower than the genetic diversity observed in studies implemented in Africa [63] or South America [51]. Comparing these works to our context, a decrease in diversity was commonly observed in other islands and especially in Martinique [64] or Dominica [51] in the Caribbean. The genetic diversity seemed to be linked with the isolation of the island, and a low level of genetic exchanges between different islands was shown in French Polynesia [25, 64]. These results were confirmed by the presence of high correlation in the Mantel test (Fig 2) and suggest that even short range mosquitoes like *Ae*. *aegypti* can disperse readily within an island, each island having its own diversity.

Our results also indicate a clear structure differentiation between New Caledonia samples and the mosquitoes of Central (FJ and TG) and East Pacific (FP) (Fig 3). It is interesting to note, that mosquito specimens collected on the same island (ie: Noumea/Poindimie and Lautoka/Suva) are more homogeneous compared to samples from different islands (ie: Ouvea, Havelu, Tubuai and Vaitahu) which are more isolated. As a fact, in 1958, *Ae*. *aegypti* was restricted to Noumea and its suburbs [65]. The first mention of this species in Ouvea was reported in 1962 [6]. Whereas travel between Noumea and Poindimie is easy by road, Ouvea has remained quite isolated from the main island, which is corroborated by the *Ae*. *aegypti*-free status of the other Loyalty islands until the end of the 1990s[66, 67].

The mosquitoes of Noumea, Suva and Papeete were more genetically mixed. New Caledonia, French Polynesia and Fiji have the largest economies in the South Pacific region [68], thus underlining the importance of the sea-ports in this specific structure. Indeed, in New Caledonia, the Nickel industry represents 75% of the export of goods: mainly to Asia, Australia and Europe. For mosquitoes collected in central Pacific islands, a differentiation was observed between Lautoka (FJ) and Havelu (TG). The genetic link between Fiji and Tonga could be explained by the relative proximity of these two island countries and the volume of trade between them. Furthermore, the goods and services importation/exportation are promoted between English or French speaking countries [12].

Environmental conditions and ecosystems could have an impact on the structure of the *Ae*. *aegypti* population. Insecticides used both for agriculture and vector control can exert specific environmental constraints. Among the different island countries, insecticide use has been implemented in different ways. In New Caledonia, malathion (organophosphate) was used until the end of the 1980s when it was replaced by deltamethrin (pyrethroid). Malathion was reintroduced in 2005 due to the detection of mosquito resistance to pyrethroids and used until present day [69]. In French Polynesia, only malathion was used before the year 2000. Malathion was then used alternatively with pyrethroids. In Tonga, malathion was used until the end of the twentieth century and was then replaced by pyrethroids. These different vector control strategies could have an impact on the genetic structure of the *Ae*. *aegypti* population [64], (due to genetic bottlenecks) along with other environmental factors (climate, human influence…).

This is, to our knowledge, the first study carried out on a Pacific scale dealing with the genetic diversity and phylogeny of *Ae*. *aegypti*. The genetic specificity could have an impact on vector competence for the arbovirus especially for dengue virus [70–72]. In the Pacific region, the arboviral outbreaks impacted island countries at different times [4]. The genetic structure in the Pacific region indicates a western, central and eastern differentiation between the *Ae*. *aegypti* samples. Previous studies reported that the vector competence of *Ae*. *aegypti* for dengue virus is linked to the mosquito genetic factor and to the dengue virus strain [72, 73]. Thus characterization of vector competence for arboviruses in Pacific island mosquitoes is also an important issue deserving investigation.

## Supporting Information

S1 Fig Factorial Correspondence Analysis of combined CO1-ND4 genes. The colour indicates the geographical sample origin: blue represents American samples, green Asian samples, purple African samples, yellow Australian samples and red Pacific samples. Both axis represent 77.9% of the variability in the dataset.
(PDF)

S1 Table Frequencies of CO1 and ND4 haplotypes for all sample sites and for each island. *N* Number of individual analyzed. Roman numerals indicate the name of CO1 or ND4 haplotypes.
(PDF)

S2 Table MtDNA haplotype sequences for CO1 and ND4 across *Ae*. *aegypti* Pacific samples. N corresponds to the number of sample belonging to this haplotype.
(PDF)

S3 Table MtDNA sequence informations of Pacific samples and reference sequences. (PDF)
