# Supplementary material for: Genetic Diversity and Phylogeny of Aedes aegypti, the Main Arbovirus Vector in the Pacific
Source: PLoS Negl Trop Dis. 2016 Jan 22;10(1):e0004374. doi: 10.1371/journal.pntd.0004374 (PMC4723151; doi:10.1371/journal.pntd.0004374)
Supplement: S1 Fig — The colour indicates the geographical sample origin: blue represents American samples, green Asian samples, purple African samples, yellow Australian samples and red Pacific samples. Both axis represent 77.9% of the variability in the dataset. (PDF) [file pntd.0004374.s001.pdf]

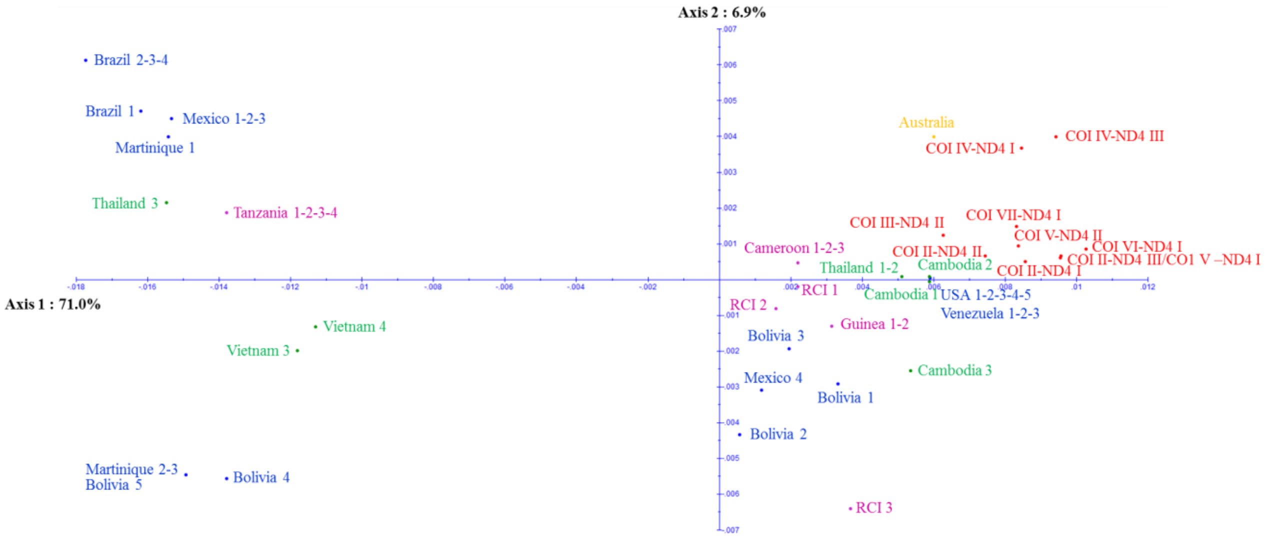

Figure S1. Factorial Correspondence Analysis of combined CO1-ND4 genes. The colour indicates the geographical sample origin: blue represents American samples, green Asian samples, purple African samples, yellow Australian samples and red Pacific samples. Both axis represent 77.9% of the variability in the dataset.
