# Supplementary material for: Genetic Diversity and Phylogeny of Aedes aegypti, the Main Arbovirus Vector in the Pacific
Source: PLoS Negl Trop Dis. 2016 Jan 22;10(1):e0004374. doi: 10.1371/journal.pntd.0004374 (PMC4723151; doi:10.1371/journal.pntd.0004374)
Supplement: S1 Table — N Number of individual analyzed. Roman numerals indicate the name of CO1 or ND4 haplotypes. (PDF) [file pntd.0004374.s002.pdf]

Table S1. Frequencies of CO1 and ND4 haplotypes for all sample sites and for each island.

Locality	*N*	CO1							*N*	ND4		
		I	II	III	IV	V	VI	VII		I	II	III
**Poi-NC**	30	-	0.50	-	0.50	-	-	-	30	0.57	-	0.43
**Nou-NC**	30	0.20	0.23	-	0.57	-	-	-	30	0.77	-	0.23
**Ouv-NC**	30	0.20	0.23	-	0.57	-	-	-	30	0.77	-	0.23
**Lau-FJ**	30	-	0.73	-	-	0.27	-	-	30	0.37	-	0.63
**Suv-FJ**	30	-	0.37	-	-	0.53	-	0.1	30	0.80	0.07	0.13
**Hav-TG**	30	0.37	-	0.63	-	-	-	-	30	0.37	0.63	-
**Tub-FP**	30	0.77	-	0.23	-	-	-	-	30	0.77	0.23	-
**Pap-FP**	30	0.60	-	0.20	-	-	0.20	-	30	0.80	0.20	-
**Vai-FP**	30	0.10	0.1	0.80	-	-	-	-	30	0.13	0.87	-
**All samples**	**270**	**0.25**	**0.24**	**0.21**	**0.18**	**0.09**	**0.02**	**0.01**	**270**	**0.59**	**0.22**	**0.19**

N Number of individual analyzed.

Roman numerals indicate the name of CO1 or ND4 haplotypes.
