# Supplementary material for: Genetic Diversity and Phylogeny of Aedes aegypti, the Main Arbovirus Vector in the Pacific
Source: PLoS Negl Trop Dis. 2016 Jan 22;10(1):e0004374. doi: 10.1371/journal.pntd.0004374 (PMC4723151; doi:10.1371/journal.pntd.0004374)
Supplement: S2 Table — N corresponds to the number of sample belonging to this haplotype. (PDF) [file pntd.0004374.s003.pdf]

Table S2. MtDNA haplotype sequences for CO1 and ND4 across *Ae. aegypti* Pacific samples.

Haplotype	N	Polymorphic positions				
**CO1**						
		298	442	556	610	703
**Haplotype I**	67	T	T	G	G	A
**Haplotype II**	65	•	•	•	•	T
**Haplotype III**	56	•	C	•	•	T
**Haplotype IV**	49	•	•	•	A	T
**Haplotype V**	24	C	•	•	•	T
**Haplotype VI**	6	•	•	A	•	•
**Haplotype VII**	3	C	C	•	•	T
**ND4**						
		188	284			
**Haplotype I**	160	G	C			
**Haplotype II**	60	•	T			
**Haplotype III**	50	A	•			

N corresponds to the number of sample belonging to this haplotype.
