# Supplementary material for: Genetic Diversity and Phylogeny of Aedes aegypti, the Main Arbovirus Vector in the Pacific
Source: PLoS Negl Trop Dis. 2016 Jan 22;10(1):e0004374. doi: 10.1371/journal.pntd.0004374 (PMC4723151; doi:10.1371/journal.pntd.0004374)
Supplement: S3 Table — (PDF) [file pntd.0004374.s004.pdf]

Table S3. MtDNA sequence informations of Pacific samples and reference sequences.

Geographical Area	Name	Number of specimens	Country	Accession Number	Reference
**CO1**					
**Pacific**	Haplotype I	12	New Caledonia	KT313642	This study
		11	Tonga	KT313643	
		44	French Polynesia	KT313644	
	Haplotype II	29	New Caledonia	KT313645	
		33	Fiji	KT313646	
		3	French Polynesia	KT313647	
	Haplotype III	19	Tonga	KT313648	
		37	French Polynesia	KT313649	
	Haplotype IV	49	New Caledonia	KT313650	
	Haplotype V	24	Fiji	KT313651	
	Haplotype VI	6	French Polynesia	KT313652	
	Haplotype VII	3	Fiji	KT313653	
			Australia	DQ026284	Beebe, *et al*, 2005
**South-America**			Bolivia 1, 2	JQ926682	Paupy, *et al*, 2012
			Bolivia 3	JQ926683	
			Bolivia 4	JQ926681	
			Bolivia 5	JQ926676	
			Brazil 1, 2, 3, 4	JQ926703	
			Venezuela 1, 2, 3	JQ926701	
**Central America**			Mexico 1, 2, 3	JQ926698	
			Mexico 4	JQ926699	
**Caribbean**			Martinique 1	JQ926696	
			Martinique 2, 3	JQ926697	
**North-America**			USA 1, 2, 3, 4, 5	JQ926684	
**South East Asia**			Cambodia 1	JQ926688	
			Cambodia 2	JQ926689	
			Cambodia 3	JQ926690	
			Thailand 1, 2	JQ926691	
			Thailand 3	JQ926692	
			Vietnam 1, 2	JQ926685	
			Vietnam 3	JQ926686	
			Vietnam 4	JQ926687	
**Africa**			Republic of Côte d'Ivoire 1	JQ926693	
			Republic of Côte d'Ivoire 2	JQ926694	
			Republic of Côte d'Ivoire 3	JQ926695	
			Tanzania 1, 2, 3, 4	JQ926704	
			Cameroon1, 2, 3	JQ926702	
			Guinea 1, 2	JQ926700	
**ND4**					
**Pacific**	Haplotype I	63	New Caledonia	KT313654	This study
		35	Fiji	KT313655	
		11	Tonga	KT313656	
		51	French Polynesia	KT313657	
	Haplotype II	2	Fiji	KT313658	
		19	Tonga	KT313659	
		39	French Polynesia	KT313660	
	Haplotype III	27	New Caledonia	KT313661	
		23	Fiji	KT313662	
**South-America**			Bolivia 1	JQ926708	Paupy, *et al*, 2012
			Bolivia 2, 3	JQ926707	
			Bolivia 4, 5	JQ926705	
			Brazil 1	JQ926718	
			Brazil 2, 3, 4	JQ926719	
			Venezuela	JQ926726	
**Central America**			Mexico 1, 2, 3	JQ926713	
			Mexico 4	JQ926714	
**Caribbean**			Martinique 1	JQ926711	
			Martinique 2, 3	JQ926712	
**North-America**			USA 1, 2, 3, 4, 5	JQ926725	
**South East Asia**			Cambodia 1, 2, 3	JQ926722	
			Thailand 1, 2	JQ926720	
			Thailand 3	JQ926721	
			Vietnam 1, 2	JQ926723	
			Vietnam 3, 4	JQ926724	
**Africa**			Republic of Côte d'Ivoire 1, 2	JQ926709	
			Republic of Côte d'Ivoire 3	JQ926710	
			Tanzania1, 2, 3, 4	JQ926715	
			Cameroon 1, 2, 3	JQ926716	
			Guinea 1, 2	JQ926717	
